# Association between markers of inflammation and indicators of systemic endotoxemia in endogenous psychosis

**DOI:** 10.1192/j.eurpsy.2022.1801

**Published:** 2022-09-01

**Authors:** S. Zozulya, I. Oleichik, I. Otman, M. Yakovlev, T. Klyushnik

**Affiliations:** 1 Mental Health Research Centre, Laboratory Of Neuroimmunology, Moscow, Russian Federation; 2 Mental Health Research Centre, Department Of Endogenous Mental Disorders And Affective Conditions, Moscow, Russian Federation; 3 Research Institute of General Pathology and Pathophysiology, Laboratory Of Systemic Endotoxemia And Shock, Moscow, Russian Federation

**Keywords:** endogenous psychosis, systemic inflammation, systemic endotoxemia, antiendotoxin antibodies

## Abstract

**Introduction:**

The clinical and biological studies indicate the involvement of inflammation in the pathogenesis of endogenous mental disorders. The inflammation markers leukocyte elastase, α1-proteinase inhibitor, and autoantibodies to neuroantigens reflect the severity of the pathological process in the brain. Systemic endotoxemia is a pathological process caused by an excess of endotoxins in the systemic circulation, can be considered as one of the components of the inflammatory process in endogenous psychosis.

**Objectives:**

To evaluate the association between systemic inflammation markers and indicators of systemic endotoxemia in patients with endogenous psychosis.

**Methods:**

The study included 25 patients aged 23-49 with endogenous psychoses (F20, F25) and 25 healthy people. The severity of symptoms was assessed using PANSS. We detected the activity of leukocyte elastase and a1-proteinase inhibitor, antibodies to neuroantigens, endotoxin (ET) concentration, and antibodies to endotoxin (aEТ) in serum.

**Results:**

In 24% of cases, an increase of inflammation markers activity, ET concentration, and aET deficiency were observed (p<0.05), which is an unfavorable factor that aggravates the clinical course of the disease. In 76% of cases, ET concentration remained within control values (p>0.05) but associated with different levels of aET, which is likely to be a consequence of previous endotoxin aggression. There were correlations between ET concentration and antibodies to neuroantigens S-100B and MBP. We also revealed the association between the activity of the inflammatory marker with the severity of clinical symptoms in patients.

**Conclusions:**

Results suggest the relationship between systemic inflammation markers and indicators of systemic endotoxemia and their involvement in the pathogenesis of endogenous psychosis.

**Disclosure:**

No significant relationships.

